# Robust phase-based texture descriptor for classification of breast ultrasound images

**DOI:** 10.1186/s12938-015-0022-8

**Published:** 2015-03-24

**Authors:** Lingyun Cai, Xin Wang, Yuanyuan Wang, Yi Guo, Jinhua Yu, Yi Wang

**Affiliations:** Department of Electronic Engineering, Fudan University, Shanghai, 200433 China; Key Laboratory of Medical Imaging Computing and Computer Assisted Intervention (MICCAI) of Shanghai, Shanghai, 200433 China; Department of Ultrasound, Huashan Hospital, Fudan University, Shanghai, 200040 China

**Keywords:** Breast ultrasound, Local binary pattern, Phase congruency, Texture feature, Tumor classification

## Abstract

**Background:**

Classification of breast ultrasound (BUS) images is an important step in the computer-aided diagnosis (CAD) system for breast cancer. In this paper, a novel phase-based texture descriptor is proposed for efficient and robust classifiers to discriminate benign and malignant tumors in BUS images.

**Method:**

The proposed descriptor, namely the phased congruency-based binary pattern (PCBP) is an oriented local texture descriptor that combines the phase congruency (PC) approach with the local binary pattern (LBP). The support vector machine (SVM) is further applied for the tumor classification. To verify the efficiency of the proposed PCBP texture descriptor, we compare the PCBP with other three state-of-art texture descriptors, and experiments are carried out on a BUS image database including 138 cases. The receiver operating characteristic (ROC) analysis is firstly performed and seven criteria are utilized to evaluate the classification performance using different texture descriptors. Then, in order to verify the robustness of the PCBP against illumination variations, we train the SVM classifier on texture features obtained from the original BUS images, and use this classifier to deal with the texture features extracted from BUS images with different illumination conditions (i.e., contrast-improved, gamma-corrected and histogram-equalized). The area under ROC curve (AUC) index is used as the figure of merit to evaluate the classification performances.

**Results and conclusions:**

The proposed PCBP texture descriptor achieves the highest values (i.e. 0.894) and the least variations in respect of the AUC index, regardless of the gray-scale variations. It’s revealed in the experimental results that classifications of BUS images with the proposed PCBP texture descriptor are efficient and robust, which may be potentially useful for breast ultrasound CADs.

## Introduction

As one of the most common cancers, breast cancer is one of the leading causes of death among women. In 2013, the estimated new cases of breast cancer were 232,340 and estimated deaths were 39,620 in the United States [1]. Because of the unknown causes of breast cancer, early detection is critical to the medical treatment [2].

Mammography is considered as one of the best available modalities for detection and diagnosis of breast cancer due to its high resolution and sensitivity, which can provide early detection for its capability of discovering micro-calcifications [2,3]. However, mammography has limitations in detecting breast cancers in women with dense breasts [4].

Currently, the ultrasound imaging has been one of the most effective and prevalent approaches for breast tumor detection for its non-invasive, no radiation and inexpensive properties. Compared with mammography, the breast ultrasound (BUS) has the ability of revealing hidden lesions in dense breast tissues. Additionally, it could distinguish benign tumors from malignant ones by characterizing their shapes, borders, internal and posterior acoustic behaviors [5]. However, the BUS diagnosis is more dependent on human expertise, and the results may be subjective and easily affected by personalized interpretation. Therefore, the computer-aided diagnosis (CAD) system is emerging as a great helper for analyzing and processing medical images, which offers more objective evaluation results and helps the radiologists to make diagnostic decisions more precisely.

Generally, an ultrasound CAD system for breast cancer is performed in four stages, including image preprocessing, lesion segmentation, feature extraction, and classification [6]. In these procedures, feature extraction of BUS images is a critical and essential stage in a CAD system. It aims to find a feature set obtained from BUS images that are accurate enough for classifying breast cancer lesions. Basically, the features of BUS images can be categorized into two classes: morphological and texture features.

Morphological features focus on the local characteristics of the mass, such as the shape and margin. Although morphological features are proven effective and commonly used in a breast ultrasound CAD [7,8], it requires the tumor contours as prior knowledge which could be obtained by the image segmentation stage. Due to the severe influence of speckles, BUS images often have drawbacks of low contrast, blurry margins and poor quality. These drawbacks make the segmentation more difficult, and therefore the segmented result is easily different with real tumor contour. These differences may directly affect the discriminant abilities of the extracted features.

Texture features depict the tissue scattering properties caused by pathological changes of the mass [6]. Unlike morphological features, most of texture features are calculated from the rough region of interests (ROIs) using the gray-level values, without the need of accurately obtained tumor contours. It has been demonstrated that texture patterns are efficient in distinguishing benign breast lesions from malignant [9,10]. Chen *et al*. [11] adopted wavelet transform to extract useful texture features from transformed BUS images and decomposition coefficients. Similarly, Mogatadakala *et al*. [12] extracted mean and variance of the order statistics after wavelet decomposition. Besides, several studies aimed to investigate useful texture features based on the gray-level co-occurrence matrix (GLCM) [9,13-17], including the contrast, the correlation and the covariance of the GLCM, and the great capability of GLCM matrix in classifying BUS images were revealed. Gómez *et al*. [18] advanced to analyze the behavior of 22 GLCM statistics with six quantization levels, four orientations and ten distances to select the most discriminative GLCM-based texture feature descriptors. In Ref. [19], Masumoto *et al*. proposed to use the local binary pattern (LBP) to extract intensity-independent and rotation-invariant texture features for classifying solid masses in BUS images. Furthermore, Yang *et al*. [20] devoted to focus on the robust texture analysis using multi-resolution gray-level invariant features via ranklet transform for breast ultrasound tumor diagnosis, and the experiments suggested the efficiency of ranklet transform-based texture features in designing a robust CAD system. Recently, contourlet-based texture analysis was also introduced for breast tumor classification by Zhang *et al.* [21]. Five texture features were extracted from the directional sub-bands after contourlet transformation, and the results demonstrated that the diagnostic performance was improved contrasted with the classic features.

Generally speaking, in BUS images, benign tumors often appear with round or ellipsoid shapes, smooth and definite borders, and homogeneous internal echoes; whereas malignant tumors often appear with irregular shapes, blurry and angular borders, inhomogeneous internal echoes. Such local structural information is actually quite significant for distinguishing benign tumors from malignant ones, and it can be precisely captured by calculating the local phase. As stated in [22], the local phase of a certain signal contains the local structural information.

Particularly, the phase information plays a more and more important role in many fields of pattern recognition in recent years. As introduced by Ref. [23,24], phase information had already been applied to texture image retrieval successfully, and the phase-based feature extraction methods were superior to some popular methods for effective image retrieval. Besides, phase information was adopted for applications related to facial recognition [25,26]. Additionally, Shojaeilangari *et al.* [27] invoked LBP method with phase information for facial expression recognition, and the results were quite promising as well. However, there is few reported research works on extracting the structural-textural features of BUS images using the phase information.

Herein, a novel phase-based texture feature descriptor with the local structural information incorporated is proposed for efficient and robust classification of BUS images. The proposed texture feature descriptor, named as the phase congruency-based binary pattern (PCBP), is an integration of the phase congruency (PC) approach [28-30] and the LBP-based method [31]. Such an integration takes advantages of both methods where the PC extracts the local structural information such as edges while the LBP extracts the local textural patterns. It’s constructed by applying the LBP variance (LBPV) method [32] on oriented PC images, which is able to capture textural patterns of the local phase information with higher discriminant ability. Thus, the proposed PCBP texture feature is an oriented local information (i.e., structural and textural) descriptor that is capable of interpreting various patterns of BUS images, and can be used in the support vector machine (SVM) for classifying BUS images. Although Ref. [27] and our work have similarity in adopting the PC approach together with the LBP-based method to construct feature descriptor, differences exist and mainly lie in two aspects. Firstly, different LBP methods are adopted for feature extraction. Instead of using the traditional LBP operator for feature encoding as Ref. [27], the proposed PCBP invokes the LBPV method, which utilizes the variance as an adaptive weight for the PCBP calculation and thereby makes the features extracted more discriminative. Secondly, the feature extraction units are also different. In Ref. [27], features are extracted block-by-block in each oriented PC image, and then concatenated sequentially to form the final feature descriptor; whereas the proposed PCBP texture features in this manuscript are extracted directly from each oriented PC image and concatenated. Thus, the feature dimension of the proposed PCBP is much lower and the computation is remarkably saved compared with the method in Ref. [27].

The main contribution of this paper is to develop a novel phase-based texture descriptor for solving the problem of differentiating benign and malignant tumors in BUS images. The proposed method creatively introduces the PC approach into BUS image analysis, which can effectively captures the important structural differences between benign and malignant tumors. Afterwards, the adoption of LBPV method makes it possible to extract the texture information from the oriented PC images in an efficient and robust way. Ultimately, the proposed PCBP texture descriptor has been established.

The remaining of this paper is organized as follows. ‘Materials’ introduces the experimental materials. ‘Methods’ describes our proposed PCBP texture descriptor for classifying BUS images in detail. In ‘Experiments and results’, the experimental results conducted on our BUS image database are illustrated. The ‘Discussions and conclusions’ is presented at last.

## Materials

In this study, the BUS image database used for experimental evaluation consists of 138 images, one for each patient, which were acquired from the Department of Ultrasound, Huashan Hospital in Shanghai, China during June 2004 to March 2005. All BUS images in database were obtained with an 8–15 MHz linear-array transducer probe equipped on an ACUSON Sequoia 512 ultrasound system. Informed consent for research use of the data was obtained from all patients in this study. Note that the BUS images were captured with the size of 768 × 576 pixels and the image pixel resolution was 0.10 mm/pixel, meaning that the size of one pixel is 0.10 mm.

From the 138 images, 69 were benign cases and 69 were malignant. All the breast tumors were hispathologically proven by fine-needle aspiration biopsy or core needle biopsy. Benign cases include fibroadenoma, adenosis and intraduct papilloma; whereas malignant cases include invasive carcinoma, ductal carcinoma in situ, intraduct papillary carcinoma and medullary carcinoma. Nevertheless, the tumors’ size are approximately in the range of 5-42 mm when considering the major axis of the tumors, with a mean value of 19.6 ± 7.8 mm.

**Figure 1:**
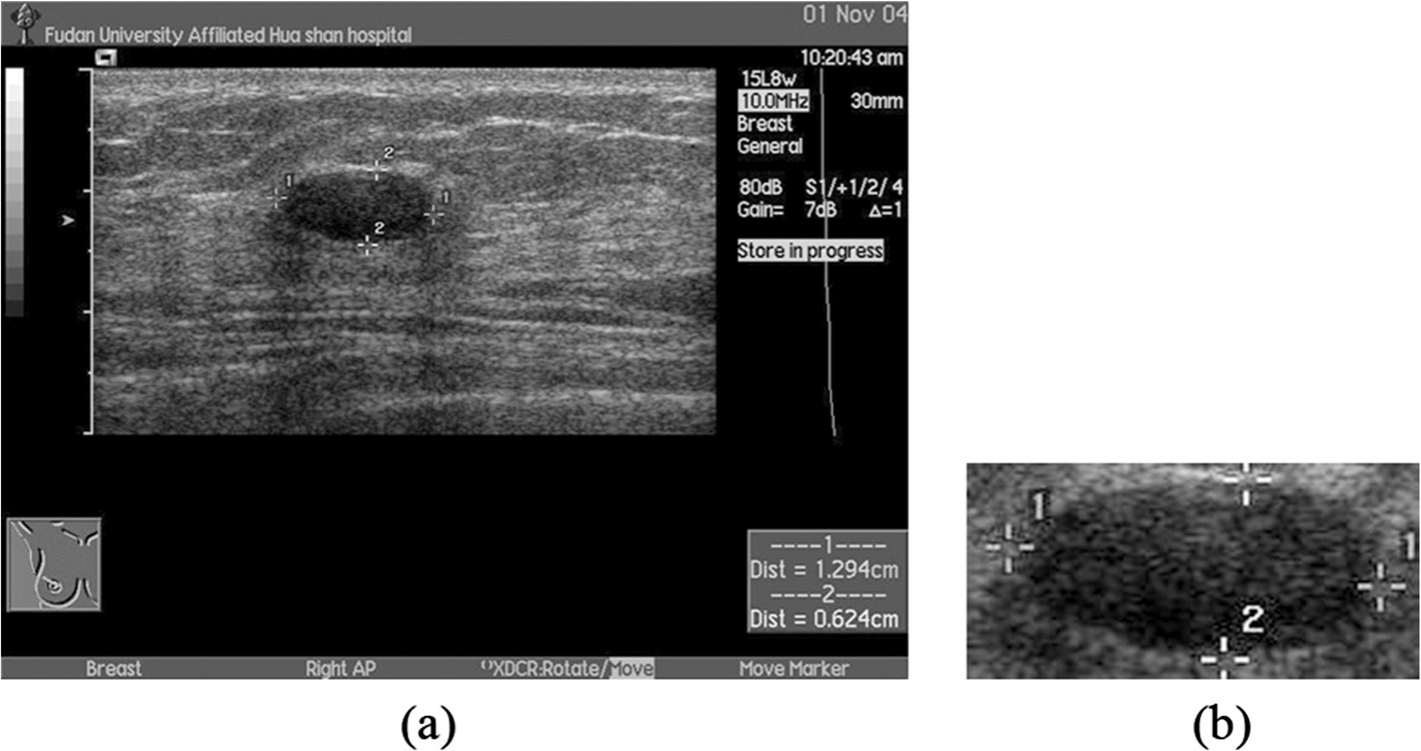
**An example of BUS images for analysis. (a)** The original BUS image; **(b)** The extracted ROI.

To obtain an accurate and effective representation of breast tumors without the trivial information such as labels, rough boundaries of tumor regions were marked by radiologists who have more than 10 years experiences of BUS examinations with four markers, which are basically at two ends of the major and minor axis of breast tumors. Then, rough ROIs are manually extracted from original BUS images based on four markers, where the tumor region located in the center position, as shown in Figure 1. Note that the sizes of rough ROIs depend on the sizes of tumors, which are between 82 × 104 pixels and 330 × 473 pixels.

## Methods

**Figure 2:**
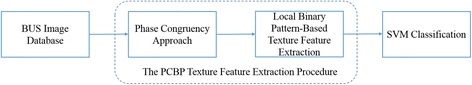
**The block diagram of the proposed texture feature analysis method.**

In this study, we focus on extracting efficient and robust texture features with the local structural information via the phase-based approach for the BUS image classification. The automatic texture feature extraction and analysis method consists of three stages, as shown in Figure 2. Firstly, the phase congruency approach is utilized to extract the local structural information on the ROIs cropped from original BUS images. Afterwards, LBP-based texture features are extracted from each oriented PC image and then merged into one concatenated feature vector, which depicts the texture information for each BUS image. Finally, the SVM is employed to differentiate benign tumors from malignant ones because of its reliable, rapid and excellent classification capability [6,8,20,33].

### Phase congruency approach

It has been shown by Oppenheim and Lim [22] that the phase information can provide more significant information within an image rather than the amplitude information. More specifically, the phase information contains the structural information (step edges, ridges, etc.) whereas the amplitude information only depicts the energy.

Any discrete signal can be represented as s sum of sine and cosine function with specific amplitudes. In the time domain, these functions form a set of scaled waves and synthesize the original signal. The phase congruency is a low-level feature detector in terms of the Fourier analysis, firstly proposed by Morrone and Owens. For a signal at the position *x*, the phase congruency is defined as [28]:

1 $$ PC(x)={ \max}_{\overline{\phi}(x)\in \left[0,2\pi \right]}\frac{{\displaystyle {\sum}_n{A}_n \cos \left({\phi}_n(x)-\overline{\phi}(x)\right)}}{{\displaystyle {\sum}_n{A}_n}}, $$

where *A*_*n*_ and *ϕ*_*n*_(*x*) represents the local amplitude and local phase angle of the *n*^th^ Fourier component, respectively. The value of $\overline{\phi}(x)$ that maximizes the Eq. (1) is the amplitude weighted mean local phase angle at the position *x*.

According to Eq. (1), it’s proven that the phase congruency is independent of the overall magnitude of the signal, thus it is consistent when the image illumination or contrast varies [28]. However, the measurement of the phase congruency in Eq. (1) has a main drawback. It does not provide good localization because it is a function of the cosine of the phase deviation. Although the cosine function is maximized when ${\phi}_n(x)=\overline{\phi}(x)$, it requires a relative significant difference between *ϕ*_*n*_(*x*) and $\overline{\phi}(x)$ before its value apparently decreases.

Inspired by the previous work, Kovesi further modified the phase congruency approach in his work [29,30] and extended it to two dimensions (2D) by calculating 1D analysis over several orientations and combined the oriented results together. In order to obtain the local frequency information, particularly the local phase, banks of filters in quadrature tuned to different spatial frequencies are required. In this study, log-Gabor filters are used [29].

Let *I* denotes a BUS image, ${M}_{s,o}^{even}$ and ${M}_{s,o}^{odd}$ denote the even-symmetric and odd-symmetric filters at the scale *s* and the orientation *o* respectively. Then the response vector can be calculated from the response of the quadrature filter pair as:

2 $$ \left[{e}_{s,o}\left(i,j\right),{o}_{s,o}\left(i,j\right)\right]=\left[I\left(i,j\right)\ast {M}_{s,o}^{even},I\left(i,j\right)\ast {M}_{s,o}^{odd}\right], $$

where ‘*’ denotes the convolution operation, (*i*, *j*) is the pixel coordinate. The amplitude of the response is:

3 $$ {A}_{s,o}\left(i,j\right)=\sqrt{e_{s,o}{\left(i,j\right)}^2+{o}_{s,o}{\left(i,j\right)}^2}, $$

and the phase is given by:

4 $$ {\phi}_{s,o}\left(i,j\right)= atan\left({o}_{s,o}\left(i,j\right)/{e}_{s,o}\left(i,j\right)\right). $$

As aforementioned, the measure of the phase congruency proposed by Morrone and Owens works poorly on localization, so a more sensitive phase deviation measure is proposed by Kovesi by incorporating the sine of the phase difference in addition to the cosine [29]:

5 $$ \varDelta {\phi}_{s,o}\left(i,j\right)= \cos \left({\phi}_{s,o}\left(i,j\right)-{\overline{\phi}}_o\left(i,j\right)\right)-\left| \sin \left({\phi}_{s,o}\left(i,j\right)-{\overline{\phi}}_o\left(i,j\right)\right)\right|. $$

Considering that the points detected by the phase congruency are significant if they occur over a wide range of frequencies, a weighting function is constructed that weakens the phase congruency at locations where the spread of the filter response is narrow [29]. For each orientation *o*, the weighting function *W*_*o*_(*i*, *j*) is defined as:

6 $$ {W}_o\left(i,j\right)=\frac{1}{1+{e}^{\alpha \left(c-{s}_o\left(i,j\right)\right)}}, $$

where *c* is the “cut-off” value of the filter response spread, and *α* is a gain factor that controls the sharpness of the cut-off. *s*_*o*_(*i*, *j*) is a fractional measure of the spread that varies between 0 to 1, which is given by:

7 $$ {s}_o\left(i,j\right)=\frac{1}{S}\left(\frac{{\displaystyle {\sum}_s{A}_{s,o}\left(i,j\right)}}{A_{\max, o}\left(i,j\right)+\eta}\right), $$

where *S* is the total number of scales, *A*_max,*o*_ is the amplitude of the maximum filter response among all scales at the orientation *o*, and *η* is a small positive constant to avoid division by zero.

Thus, the measure of the phase congruency at the orientation *o* can be defined as:

8 $$ P{C}_o\left(i,j\right)=\frac{{\displaystyle {\sum}_s{W}_o\left(i,j\right)}\left\lfloor {A}_{s,o}\left(i,j\right)\varDelta {\phi}_{s,o}\left(i,j\right)-{T}_o\right\rfloor }{{\displaystyle {\sum}_s{A}_{s,o}\left(i,j\right)}+\varepsilon }, $$

where ‘⌊ ⋅ ⌋’ denotes zeroing of negative values, *T*_*o*_ is the orientation-specific noise compensation term [30] and *ε* is a small positive constant to avoid zero denominator.

**Figure 3:**
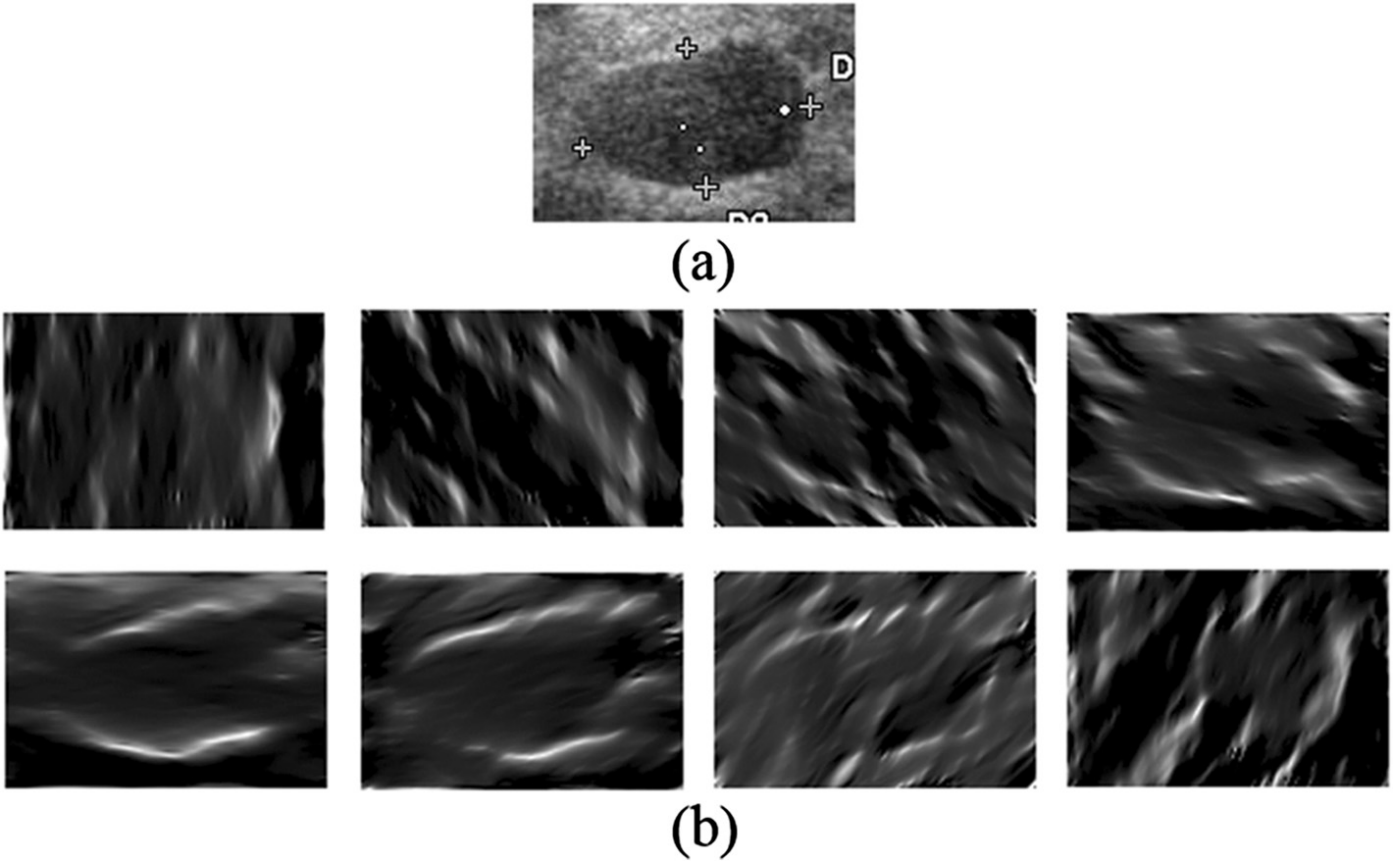
**Results of the phase congruency approach applied to a BUS image. (a)** The original BUS image; **(b)** Eight oriented PC images.

In this study, the parameters for phase congruency approach are set as follows, according to [29]. The cut-off value *c* of the weighting function in Eq. (6) is set to 0.4 and the gain factor *α* is set to 10. Both *η* and *ε* are set to 0.0001. If we apply the phase congruency approach to BUS images over six scales and eight orientations, as a result, eight oriented PC images are obtained for each BUS image, as shown in Figure 3. The *PC*_*o*_ takes value in [0, 1], which suggests smooth regions with a small value and potential boundaries with a big value. It’s clearly shown in Figure 3(b) that each PC image is calculated along a specific orientation, uniformly changing from –π/2 to π/2. In order to combine the oriented phase congruency information together for an overall understanding, the total sum of the *PC*_*o*_ over all orientations is performed.

**Figure 4:**
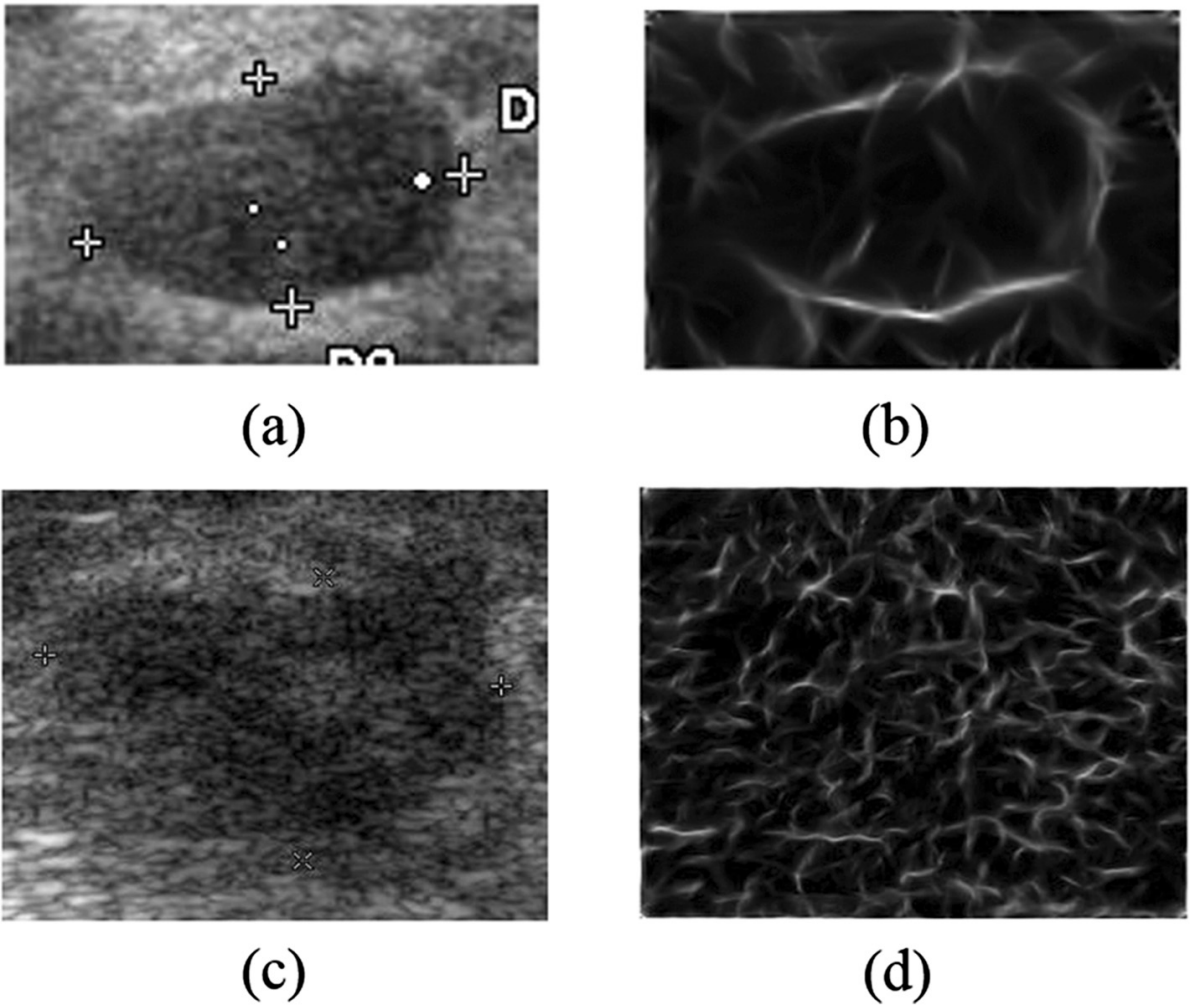
**Overall phase congruency results of BUS images. (a)** The benign tumor; **(b)** The overall phase congruency of (a); **(c)** The malignant tumor; **(d)** The overall phase congruency of **(c)**.

It is shown in Figure 4 that the overall phase congruency results of both benign and malignant cases in BUS images. Benign tumors often possess the characteristics of regular shape, clearly-defined boundaries and homogeneous internal echoes, whereas malignant tumors appear with irregular shapes, blurry and angular borders, inhomogeneous internal echoes in BUS images. These structural properties can be well reflected on the overall PC image. As shown in Figure 4(b), most of the in-phase points are located around the tumor boundary while the rest remain almost zero, indicating that the tumor has relatively well-defined boundary and smooth foreground/background regions. However, Figure 4(d) shows a different result, where most of the in-phase points are disorganized and dispersed, without a clearly illustration of the tumor location compared with the result in Figure 4(b), which are consistent with the properties of malignant tumors in BUS images. Herein, the phase congruency possesses great potentials and capabilities in depicting differences of benign and malignant tumors in BUS images via structural properties.

### Local binary pattern (LBP)-based texture feature extraction

In order to extract texture patterns from oriented PC images to construct the proposed PCBP texture descriptor, the LBP-based method is employed. The LBP [31] is a nonparametric gray-scale texture descriptor, which effectively characterizes the spatial structure of the local image textures by comparing each pixel with its neighboring pixels. Thus, given a central pixel *c* with the coordinate (*i*, *j*) in an oriented PC image *PC*_*o*_, an initial PCBP pattern code can be computed as:

9 $$ PCB{P}_{P,R,o}^{initial}\left(i,j\right)={\displaystyle \sum_{p=0}^{P-1}s\left({g}_p-{g}_c\right){2}^p},s(x)=\left\{\begin{array}{c}\hfill 1,x\ge 0\hfill \\ {}\hfill 0,x<0\hfill \end{array}\right., $$

where *g*_*c*_ and *g*_*p*_ are the gray-level values of the central pixel and *P* surrounding pixels in the circle neighborhood with a radius *R*, respectively. This comparison leads to a circular binary sequence representation of the neighbor pixels.

Uniformity of the initial PCBP pattern is defined by calculating the number of spatial transitions (from 0 to 1 or from 1 to 0) in that pattern:

10 $$ U\left(PCB{P}_{P,R,o}^{initial}\left(i,j\right)\right)=\left|s\left({g}_{P-1}-{g}_c\right)-s\left({g}_0-{g}_c\right)\right|+{\displaystyle \sum_{p=1}^{P-1}\left|s\left({g}_{p-1}-{g}_c\right)-s\left({g}_{p-1}-{g}_c\right)\right|}, $$

The pattern is considered “uniform” if the *U* value is no more than 2, which has been proved as fundamental patterns of the local image texture [31,34]. Furthermore, the rotation invariant property is taken into consideration for a more robust PCBP texture descriptor. If the patterns are equal when circularly rotated, they will be regarded same. So a local rotation invariant uniform (denoted as *riu*2) PCBP pattern can be further defined as:

11 $$ PCB{P}_{P,R,o}^{riu2}\left(i,j\right)=\left\{\begin{array}{c}\hfill {\displaystyle \sum_{p=0}^{P-1}s\left({g}_p-{g}_c\right)\kern1em \mathrm{if}\ U\left(PCB{P}_{P,R,o}^{initial}\right)\le 2}\hfill \\ {}\hfill P+1\kern3.25em \mathrm{otherwise}\hfill \end{array}\right., $$

which dramatically reduces the number of PCBP patterns from 2^*P*^ to (*P*+2).

Since the $PCB{P}_{P,R,o}^{riu2}$ only considers the spatial pattern but ignores the image contrast, a complementary local variance measure *VAR* [31] is defined on the circle neighborhood in addition, which incorporates the local contrast of images and is given by:

12 $$ VA{R}_{P,R,o}\left(i,j\right)=\frac{1}{P}{\displaystyle \sum_{p=0}^{P-1}{\left({g}_p-u\right)}^2},u=\frac{1}{P}{\displaystyle \sum_{p=0}^{P-1}{g}_p}. $$

To combine the $PCB{P}_{P,R,o}^{riu2}$ and *VAR*_*P*,*R*,*o*_ together for a better characterization of the local texture, the LBPV method [32] proposed by Guo *et al*. is adopted in our study. The core idea of the LBPV is to use the variance *VAR*_*P*,*R*,*o*_ as an adaptive weight for each $PCB{P}_{P,R,o}^{riu2}$ pattern in the histogram calculation, because it has been verified that the regions with high frequency textures will have higher variance values and they make more contributions to the discrimination of texture images [35]. Supposing the oriented PC image *PC*_*o*_ is in size of *M* × *N* and both the $PCB{P}_{P,R,o}^{riu2}$ and *VAR*_*P*,*R*,*o*_ patterns have been calculated for each pixel (*i*, *j*), a joint PCBP pattern histogram for representing *PC*_*o*_ is computed as:

13 $$ PCB{P}_{P,R,o}^{jo\mathrm{i}\mathrm{n}\mathrm{t}}(k)={\displaystyle \sum_{i=1}^M{\displaystyle \sum_{j=1}^Nw\left(PCB{P}_{P,R,o}^{riu2}\left(i,j\right),k\right),\kern0.5em k\in \left[0,K\right]}}, $$

14 $$ w\left(PCB{P}_{P,R,o}^{riu2}\left(i,j\right),k\right)=\left\{\begin{array}{c}\hfill VA{R}_{P,R,o}\left(i,j\right),\kern0.75em PCB{P}_{P,R,o}^{riu2}\left(i,j\right)=k\hfill \\ {}\hfill 0\kern5em \mathrm{otherwise}\hfill \end{array}\right., $$

where *K* is the maximum $PCB{P}_{P,R,o}^{riu2}$ pattern value.

After the joint histograms $PCB{P}_{P,R,o}^{joint}$ for each oriented PC image *PC*_*o*_ are obtained, these $PCB{P}_{P,R,o}^{joint}$ histograms are then normalized and concatenated sequentially to form the proposed PCBP texture descriptor *PCBP*_*P*,*R*_. Larger *R* and *P* will make it possible to take more local detail information of the BUS images into account when extracting features, thus may lead to a better classification performance. However, this will also make it more time-consuming. In this study, for ease of calculation, we adopt the simplest way, that is, 8 surrounding pixels (*P* = 8) with 1-pixel radius (*R* = 1) to calculate the PCBP texture descriptor as default setting, which means 10 *PCBP*^*joint*^ textures are obtained from each oriented *PC*_*o*_ image. Suggest the total numbers of scales and orientations are defined as *S* and *O*, respectively. Therefore, a feature set including (10 × *O* orientations) textures is produced as the texture representation of each BUS image. Details of the parameter selection for PCBP texture descriptor are addressed in subsection ‘Parameter selection of the PCBP texture descriptor’ later, in terms of the numbers of scales *S* and orientations *O*.

### Texture feature classification with support vector machine

Features extracted by the preceding method are then fed into classifiers to verify the efficiency for distinguishing benign and malignant tumors in BUS images. From Fisher linear discriminant analysis (FLDA) [18,36] to artificial neural networks (ANN) [11,37,38], many classifiers have been successfully applied for classifications of BUS images. Among these, the SVM is widely used due to its fast and high generalization performance [6,8]. When dealing with high dimensional data, the kernel functions are utilized to map the input feature data into higher dimension for better distributions between two classes (i.e. benign and malignant).

In this study, we use a nonlinear SVM with the radial basis function (RBF) kernel as our classifier. Before training the SVM classifiers, each feature space is scaled to the same range of [−1, 1]. The parameters of SVM, namely the regularization parameter *C* and kernel parameter *γ*, are critical to the classification performances, and the best parameters for one feature space are not necessarily the best for another feature space. Besides, they’re also actually very important for reducing the impact of over-fitting, since *C* controls the tradeoff between the training error and model complexity, which ultimately aims to fit the training data and avoiding over-fitting [20]. To effectively deal with the parameter selection problem for SVM classifiers, grid search is applied to determine the best SVM parameters, as suggested in Ref. [39]. For a specific feature space, the best parameters can be determined by employing *k*-fold cross-validation (*k* = 10) on the training data with varied parameter settings, and parameters corresponding to the best classification performance would be chosen to construct the optimal SVM model. Note that all the SVM classification procedures are implemented by utilizing the LIBSVM package [40].

Several evaluation criteria are used to quantitatively assess the diagnostic performance of the SVM classification. A receiver operating characteristic (ROC) curve [41] is most frequently used because of its comprehensive evaluation ability. The area under the ROC curve (AUC) can be used as a criterion of the overall performance for the SVM classification. The AUC value locates within [0, 1] where unity stands for ideal classification. Other criteria include the accuracy (ACC), the sensitivity (SENS), the specificity (SPEC), the positive predictive value (PPV), the negative predictive value (NPV) and the Matthew’s correlation coefficient (MCC), which are defined as [6]:

15 $$ ACC=\frac{TP+TN}{TP+TN+FP+FN} $$

16 $$ SENS=\frac{TP}{TP+FN} $$

17 $$ SPEC=\frac{TN}{TN+FP} $$

18 $$ PPV=\frac{TP}{TP+FP} $$

19 $$ NPV=\frac{TN}{TN+FN} $$

20 $$ MCC=\frac{TP\times TN-FP\times FN}{\sqrt{\left(TP+FP\right)\left(TP+FN\right)\left(TN+FP\right)\left(TN+FN\right)}} $$

where *TP* and *FN* refer to the number of correctly and incorrectly classified malignant tumors, while *TN* and *FP* indicate the number of correctly and incorrectly classified benign tumors, respectively. For the abovementioned six criteria, higher values indicate better classification performances. Note that the MCC [42,43] is a powerful criterion for accuracy evaluation, which takes value in [−1, +1] with +1 representing a perfect prediction. When the number of negative samples and positive samples are obviously unbalanced, the MCC gives a better evaluation than the accuracy [6].

## Experiments and results

In this section, three state-of-art texture feature extraction methods are employed for the performance comparison with the proposed PCBP texture descriptor, denoted as *PCBP*. The methods for comparison are ranklet transform-based texture features [20], GLCM-based texture features [18] and LBP-based texture features [19], denoted as *Ranklet*, *GLCM* and *LBP* for simplicity, respectively.

The parameters for each compared feature extraction method are set as below. Note that texture features corresponding to the best classification performance are extracted, as concluded in [18-20], and the extracted features are directly used for classification without selection. More specifically, for *Ranklet*, each BUS image is decomposed into two resolutions and corresponding three orientations via ranklet transform, then 12 GLCM-based texture codes are extracted from each ranklet transformed images and finally 72 (i.e., 2 resolutions × 3 orientations × 12 texture codes) features are obtained for texture representation, as suggested in Ref. [20]. For *GLCM*, 17 GLCM features in different orientations and distances with 32 quantization levels are selected as texture representation. Details can be referred to Ref. [18]. For *LBP*, LBP patterns with 24 neighbor pixels (*P* = 24) and 3-pixel radius (*R* = 3) are calculated and 26 features are set as the texture representation, according to Ref. [19]. Thus, the feature dimensions of *Ranklet*, *GLCM* and *LBP* are 72, 17 and 26, respectively. Experiments are conducted to clarify the issue about parameter selection for the *PCBP* texture descriptor firstly, then to verify the efficiency and robustness respectively, followed by statistical analysis and computation time evaluation.

### Parameter selection of the PCBP texture descriptor

**Figure 5:**
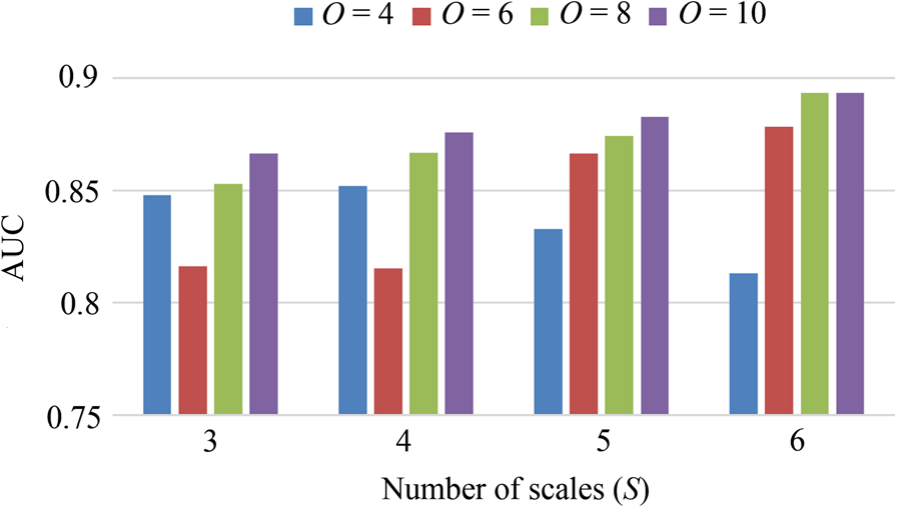
**Classification performance (AUC values) of** ***PCBP*** **texture descriptor using different numbers of scales (** ***S*** **) and orientations (** ***O*** **).**

To clarify the issue about parameter selection for the proposed *PCBP* texture descriptor (i.e. numbers of orientations *O* and scales *S*), firstly, we carry out the experiments with four orientations (i.e., *O* = {4, 6, 8, 10}) and four scales (i.e., *S* = {3, 4, 5, 6}) that are commonly used for PC calculation [29,30]. Feature spaces corresponding to different combinations of *O* and *S* are produced and then sent into SVM classifiers. Because a relatively small database (i.e., including 138 cases) is used for experiments, we adopt the leave-one-out cross-validation (LOO-CV) method to evaluate the classification performance. As the name suggests, the LOO-CV involves using only one case as testing data while the remaining cases as training data. This process is repeated until every case in database is used once as the testing data. Note that for each feature space, grid search with cross-validation is applied for constructing the optimal SVM classifier. The AUC values obtained from LOO-CV method are used as the figure of merit, which can give comprehensive evaluations for the classification performances. The results are depicted in Figure 5.

Generally speaking, the AUC value is growing as both the numbers of scales *S* and orientations *O* are getting larger, as shown in Figure 5. The highest AUC value (i.e., 0.894) is achieved in the situations where the number of scales is six and number of orientations is either eight (i.e., bar in green) or ten (i.e., bar in purple). Recall that the feature dimension for the *PCBP* texture descriptor is related to the number of orientations (i.e., 10 × *O* orientations). Larger *O* indicates higher dimensions of feature space, as a consequence, there is a stronger chance that the SVM classifier would over-fit the data with limited samples (i.e., 138 cases) [20]. Besides, feature space with higher dimension is more time-consuming when calculated. Therefore, in this study, the proposed *PCBP* texture descriptor is calculated over six scales (*S* = 6) and eight orientations (*O* = 8) to avoid the abovementioned problems, and finally 80 (i.e. 10 × 8 orientations) features are extracted as texture representation for each BUS image.

### Efficiency of the PCBP texture descriptor for classification

To verify the efficiency of the proposed texture descriptor for classification, all features including *Ranklet*, *GLCM*, *LBP* and the proposed *PCBP* are extracted from the same database and then fed into SVM classifiers separately. The quantitative evaluation results with the LOO-CV method are detailed listed in Table 1.

**Table 1:** **Performance evaluation of** ***Ranklet*** **,** ***GLCM*** **,** ***LBP*** **and** ***PCBP*** **with the LOO-CV method**

**Methods**	**AUC**	**ACC (%)**	**SENS (%)**	**SPEC (%)**	**PPV (%)**	**NPV (%)**	**MCC**
*Ranklet*	*0.882*	81.16	73.91	**88.41**	*86.44*	77.22	0.630
*GLCM*	0.848	77.54	68.12	*86.96*	83.93	73.13	0.561
*LBP*	0.850	*83.33*	*81.16*	85.51	84.85	*81.94*	*0.667*
*PCBP*	**0.894**	**86.96**	**86.96**	*86.96*	**86.96**	**86.96**	**0.739**

Note. The best performance for each criterion is highlighted with bold, and the second best is italic.

From Table 1, it’s noticeable that the performance of the proposed *PCBP* achieves the best in AUC value, which demonstrates the discrimination ability of the *PCBP* from a comprehensive view. Moreover, the performance of the *PCBP* ranks the first in five criteria out of the remaining six, especially in terms of the SENS, NPV and MCC values, the improvements are 5% ~ 10% or more when compared with the second best results.

As reference, Table 2 gives the SVM classifier parameters *C* and *γ* for each texture descriptor selected by grid search. For objectively comparing the classification performances, these parameters are fixed for each specific texture descriptor over the rest of the experiments.

**Table 2:** **SVM classifier parameters for** ***Ranklet*** **,** ***GLCM*** **,** ***LBP*** **and** ***PCBP***

**Parameters**	***Ranklet***	***GLCM***	***LBP***	***PCBP***
*C*	1024	0.5000	2.8284	2
*γ*	0.0110	0.0442	1	0.0884

Considering the performance evaluation with the LOO-CV method might be upward bias [44], we adopt the bootstrap method (as also used in Ref. [18] and Ref. [20]) with 500 independent bootstrap samples to evaluate the classification performance as well. For each bootstrap sample, the training data are built by randomly resampling the database with replacement until the size of the training data is the same as that of the database, whereas the testing data are selected as those not included in the training data. To be more specific, we present the performance evaluations with 500 independent bootstrap samples in Table 3.

**Table 3:** **Performance evaluations of** ***Ranklet*** **,** ***GLCM*** **,** ***LBP*** **and** ***PCBP*** **with bootstrap method (mean ± standard deviation)**

**Methods**	**AUC**	**ACC (%)**	**SENS (%)**	**SPEC (%)**	**PPV (%)**	**NPV (%)**	**MCC (%)**
*Ranklet*	0.843 ± 0.044	80.68 ± 5.55	76.68 ± 10.86	**85.39 ± 7.24**	83.45 ± 7.90	78.61 ± 9.57	0.625 ± 0.105
*GLCM*	0.832 ± 0.043	78.40 ± 4.80	75.17 ± 11.35	82.36 ± 10.12	81.67 ± 8.83	77.39 ± 9.11	0.583 ± 0.094
*LBP*	0.807 ± 0.048	77.77 ± 5.53	76.27 ± 10.03	79.87 ± 9.26	79.10 ± 8.95	77.61 ± 8.50	0.564 ± 0.108
*PCBP*	**0.862 ± 0.037**	**83.17 ± 4.81**	**83.36 ± 7.64**	83.42 ± 8.32	**84.25 ± 7.24**	**83.58 ± 7.23**	**0.670 ± 0.094**

Note. The best performance for each criterion is highlighted with bold.

**Figure 6:**
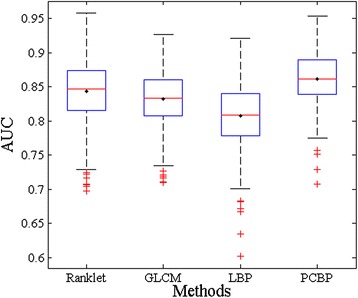
**Boxplots of AUC values calculated from 500 independent bootstrap samples.** The black point in each box indicates the mean value.

As shown in Table 3, the classification performances with the bootstrap method remain consistent with the results given by Table 1. In Table 3, with respect to both the mean value and standard deviation, the classification performance of the proposed *PCBP* achieves the best in six criteria, but a little lower in SPEC than that of the *Ranklet*. Since the ROC analysis has a relatively objective evaluation for the classification performance, we adopt the AUC values calculated from 500 independent bootstrap samples for boxplots to visualize the discrimination power of each texture descriptor. As depicted in Figure 6, the median values of the AUC for *Ranklet*, *GLCM*, *LBP* and *PCBP* are 0.848, 0.834, 0.809 and 0.861 respectively, which are quite similar to their mean values. It’s observed that the proposed *PCBP* texture descriptor performs much better and more stable for classification, providing higher median value (i.e., 0.861), smaller dispersion range (i.e., from 0.775 to 0.954) and less outliers (i.e., 4).

### Robustness of the PCBP texture descriptor for classification

Majority of the existing studies for the texture classification of BUS images [9-12] are performed under the assumption that the gray-scale range of an image to be classified is consist with those in the training set. However, with respect to the practical applications, it is usual that BUS images are captured under different illumination conditions due to the adjustable parameters of ultrasonic devices [20]. Thus, BUS images would be in different gray-scale range under different situations. In order to verify the robustness of the proposed *PCBP* texture descriptor against gray-scale variations, experiments with variable contrast settings are conducted.

**Figure 7:**
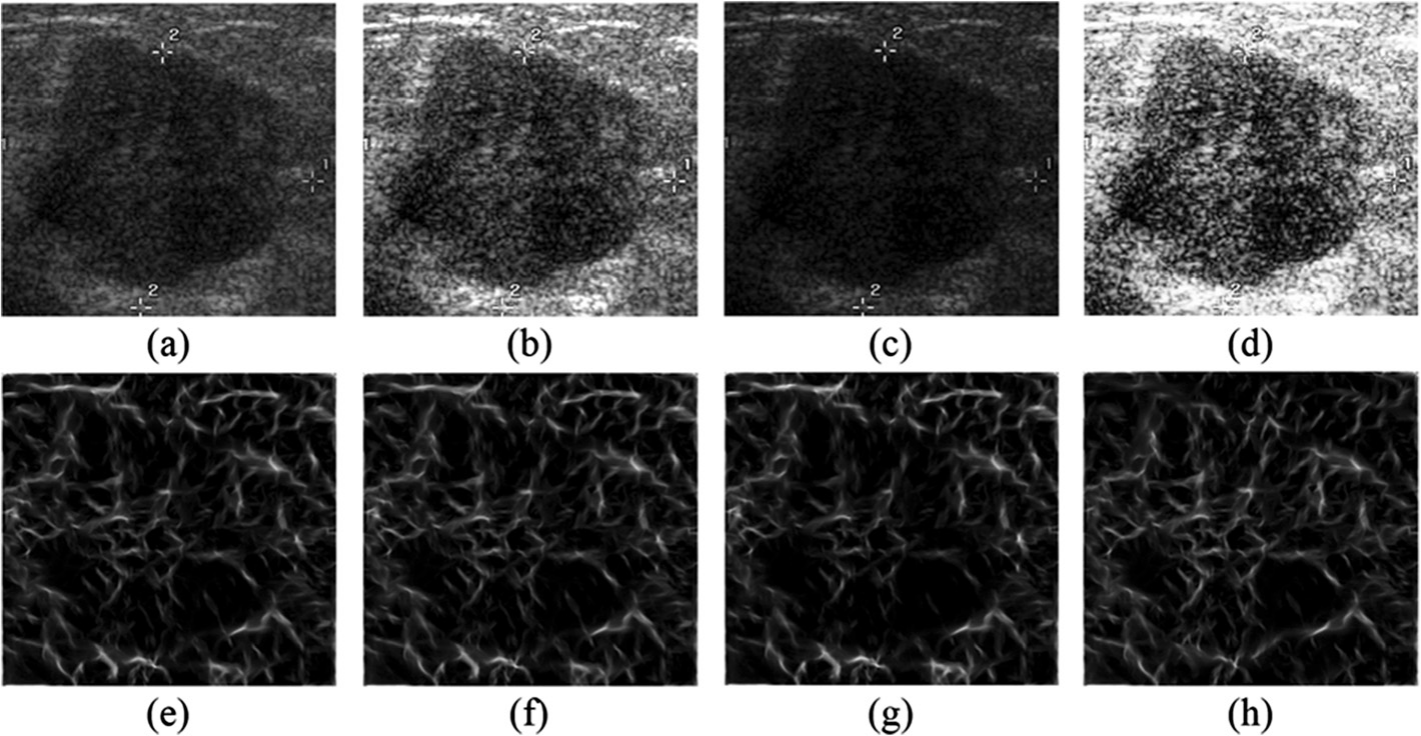
**A BUS image with varied contrast settings. (a)** The original BUS image; **(b)** The contrast-improved image; **(c)** The gamma-corrected image; **(d)** The histogram-equalized image; **(e) ~ (h)** The corresponding overall PC images of (a) ~ (d), respectively.

One linear monotonic gray-scale transformation and two nonlinear monotonic gray-scale transformations, namely the contrast improvement (CI), the gamma correction (GC) and the histogram equalization (HE) are applied to the original BUS image database to change the illumination artificially. Contrast improvement linearly maps the gray-scale values of the processed image to a new range; gamma correction, however, non-linearly maps the gray-scale values of the processed image by a power-law function; histogram equalization is a non-linear gray-scale transformation as well, which spreads the distribution of the gray-scale values evenly over the entire range [45]. The gray-scale transformed databases are denoted as *CI*, *GC* and *HE* for simplicity. The phase congruency approach is performed to derive PC images from the original BUS image and three gray-scale transformed images respectively, as depicted in Figure 7. To illustrate the theoretical contrast-invariant property of the phase congruency, instead of presenting eight oriented PC images separately, we present the corresponding overall PC image, which is the total sum of oriented PC images. From Figure 7, it’s observed that overall PC images derived from an original BUS image or enhanced images basically remain consistent in the corresponding tumor region of the BUS image. In other words, with the solid foundation constructed by the phase congruency approach, we can extract the *PCBP* texture descriptor from oriented PC images, which are invariant to gray-scale variations. It is proven that the proposed *PCBP* descriptor is able to provide robust classification performances for BUS images.

**Figure 8:**
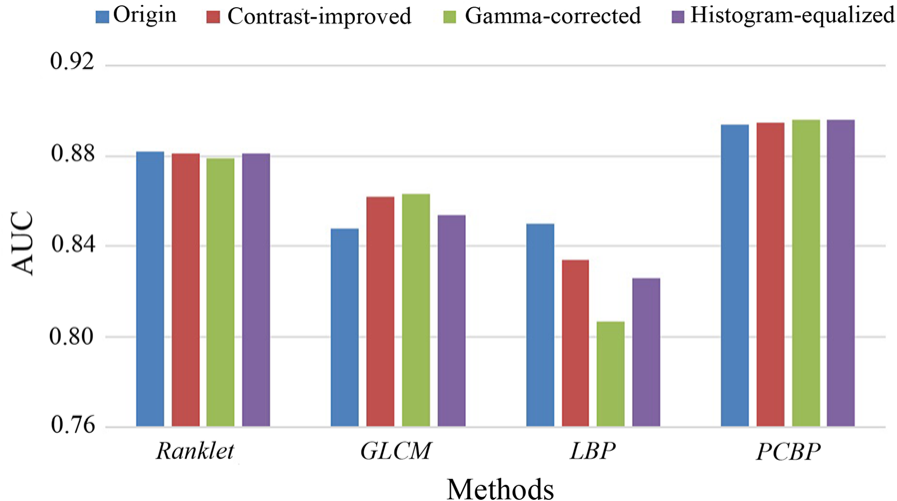
**The performance evaluation (AUC values) of the cross-contrast training/testing scheme with the LOO-CV method.**

Afterwards, texture features are extracted from the *CI*, *GC* and *HE* databases, respectively. A cross-contrast training/testing scheme is then employed for performing the classification. In this scheme, the training phase is carried out on the original BUS image database while the testing phase is performed on the gray-scale transformed databases (i.e., *CI*, *GC* and *HE* respectively), excluded in the training phase. The AUC index is used as the figure of merit for evaluation. Experiments are firstly conducted using LOO-CV method, and the results are illustrated in Figure 8. in form of bar plot. It is noted that AUC values presented in last subsection (i.e., in Table 1 and Table 3) are also included as *Origin*, and AUC values related to the *Origin*, *CI*, *GC*, *HE* databases are expressed as {*Origin*, *CI*, *GC*, *HE*} for simplicity.

As shown in Figure 8, the blue, red, green and purple bars represent the AUC values derived from the *Origin*, *CI*, *GC* and *HE* databases by each method, respectively. In terms of the AUC values, the classification performances of the *PCBP*, which are {0.894, 0.895, 0.896, 0.896}, achieve the best no matter which gray-scale transformed database is selected for testing. Furthermore, the variations of AUC values among databases are very small as well. Ranklet transformed images are also claimed to be very robust against gray-scale variations, since ranklet transformation deals with the rank of pixels rather than their gray-scale intensities [20,46]. Therefore, *Ranklet* also performs well when dealing with linear/nonlinear monotonic gray-scale variations, and the results are {0.882, 0.881, 0.879, 0.881}. As for *GLCM* and *LBP*, the performances are regarded as less effective, taking both the AUC value and its variations into consideration. *GLCM* obtains performance upgrades with the testing phase carried on all three databases, whereas the *LBP* suffers from performance degradations, suggesting that the *LBP* is less effective in handling both linear and nonlinear monotonic gray-scale variations.

**Figure 9:**
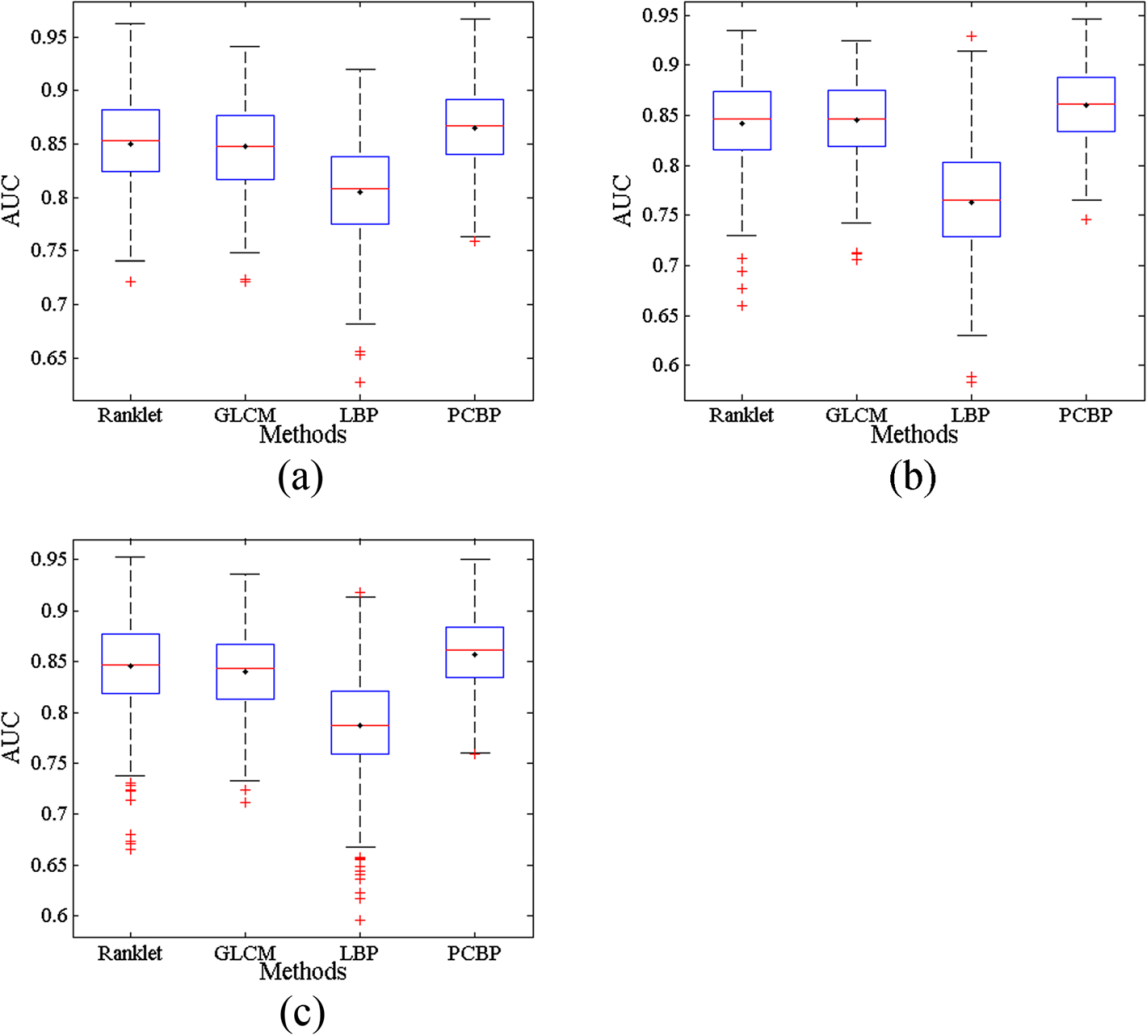
**Boxplots of AUC values obtained with the bootstrap method after employing cross-contrast training/testing scheme.** The black point in each box indicates the mean value. **(a)** Contrast-improved; **(b)** Gamma-corrected; **(c)** Histogram-equalized.

Experiments are also conducted using bootstrap method. Table 4 gives AUC values calculated from 500 independent bootstrap samples when adopting the cross-contrast training/testing scheme for the classification. Similarly, the performance evaluations of the proposed *PCBP* outperform those of other methods with the highest AUC values and smallest standard deviations. Nevertheless, the corresponding boxplots of AUC values obtained from bootstrap samples are depicted in Figure 9, where the *PCBP* achieves the highest median value, smallest dispersion range and least outliers in all three different gray-scale transformed situations.

**Table 4:** **The performance evaluation (the AUC value) of the cross-contrast training/testing scheme with the bootstrap method (mean ± standard deviation)**

**Methods**	***Origin***	***CI***	***GC***	***HE***
*Ranklet*	0.843 ± 0.044	0.850 ± 0.042	0.842 ± 0.044	0.845 ± 0.045
*GLCM*	0.832 ± 0.043	0.848 ± 0.040	0.845 ± 0.039	0.840 ± 0.040
*LBP*	0.807 ± 0.048	0.805 ± 0.047	0.763 ± 0.054	0.787 ± 0.051
*PCBP*	**0.862 ± 0.037**	**0.865 ± 0.037**	**0.861 ± 0.035**	**0.857 ± 0.038**

Note. The best performance for each criterion is highlighted with bold.

### Statistical analysis

Even though that both the ROC analysis and boxplots are effective ways for visualizing the discrimination power of texture features, it is also important to conduct the statistical analysis to make more objective evaluations. Thus, we use AUC values generated by the bootstrap method (i.e., Table 4) to perform the statistical analysis. The results obtained by the cross-contrast training/testing scheme are also included.

The statistical analysis is conducted in two experiments: 1) determining the differences in AUC values between the *PCBP* and the three compared texture descriptors (i.e., *Ranklet*, *GLCM* and *LBP*) in each database and 2) evaluating the differences in AUC values between *Origin* database and other three gray-scale transformed databases (i.e., *CI*, *GC* and *HE*) of each texture descriptor, which can statistically verify the classification efficiency and robustness of the proposed *PCBP* texture descriptor.

Instead of performing the significance tests directly, the Shapiro-Wilk test is firstly applied to test the distribution normality of AUC values obtained from each evaluated group. Since AUC values for all groups present normal distribution, the *F*-test is further used to verify whether two different groups have the same variance. It is found that the variances between data of different texture descriptors at the same database are unequal, whereas the variances between data of different databases for one texture descriptor are equal. Hence, to carry out the first experiment we employ the Welch’s *t*-test and for the second experiment we use the Student’s *t*-test.

Concerning that both experiments depicted previously involve multiple testing, Bonferroni correction is performed to account for Type I error [47]. Recall that the *p*-value less than 0.05 indicates statistical significance when considering a single comparison only. Three significant tests are made on the basis of one compared data, and therefore, the corrected significant value for *p* is set as 0.0167 (i.e., 0.05/3). Details of the statistical analysis are shown in Table 5 and Table 6, in terms of the *p*-value.

**Table 5:** **The** ***p*** **-value of the Welch’s** ***t*** **-test for determining differences in AUC values between the** ***PCBP*** **and other methods at the** ***Origin*** **,** ***CI*** **,** ***GC*** **and** ***HE*** **databases**

**Methods**	***Origin***	***CI***	***GC***	***HE***
*PCBP* vs. *Ranklet*	4.53e-13*	8.12e-9*	2.63e-13*	8.56e-6*
*PCBP* vs. *GLCM*	3.32e-30*	1.82e-12*	3.31e-11*	2.37e-12*
*PCBP* vs. *LBP*	1.98e-75*	1.37e-89*	9.88e-169*	9.50e-106*

Note. *indicates the performance difference between two methods is statistically significant.

**Table 6:** **The** ***p*** **-value of the Student’s** ***t*** **-test for evaluation differences in AUC values between the** ***Origin*** **and other databases of the** ***PCBP*** **,** ***Ranklet*** **,** ***GLCM*** **and** ***LBP***

**Databases**	***Ranklet***	***GLCM***	***LBP***	***PCBP***
*Origin* vs. *CI*	8.57e-3	1.01e-08	4.39e-01 †	2.35e-1 †
*Origin* vs. *GC*	6.72e-1 †	8.24e-07	5.83e-36	5.83e-1 †
*Origin* vs. *HE*	4.37e-1 †	2.50e-03	8.16e-10	3.56e-2 †

Note. † indicates no statistical difference is observed for the comparison.

On one hand, it’s notable from Table 5 that all the *p*-values listed are far less than the significant level 0.0167 (i.e., 1.67e-2), and we can conclude that the proposed *PCBP* statistically outperforms the *Ranklet*, *GLCM* and *LBP* in the classification efficiency of BUS images, regarding all comparisons presented in Table 5.

On the other hand, in Table 6, no statistical differences are observed for *PCBP* when comparisons of AUC values between *Origin* and other databases are performed, since all the *p*-values are greater than 1.67e-2. As for *Ranklet*, statistical difference is occurred in the comparison between AUC values of the *Origin* and *CI* databases, while no differences are shown in the rest two comparison groups. With respect to *GLCM* and *LBP*, the statistical differences of classification performances between varied contrast settings are relatively significant. The conclusions derived from Table 6 is consistent with those from last subsection, which demonstrate the robustness of the proposed *PCBP* texture descriptor against gray-scale variations for BUS image classification in a statistical perspective.

### Computation time analysis

In addition to the classification performances presented above, the computation time for extracting each feature descriptor (i.e., *Ranklet*, *GLCM*, *LBP* and *PCBP*) is also taken into consideration for the evaluation. For fair comparison, all the algorithms were performed on the same BUS image database and executed on the same platform (MATLAB R2010b, 2.10-GHz Intel Xeon E5 CPU). Average time cost of each method is given in Table 7.

**Table 7:** **The average time cost comparison**

**Methods**	***Ranklet***	***GLCM***	***LBP***	***PCBP***
Average time cost (s)	69.83	0.10	212.80	0.79

As listed in Table 7, although the average time cost for the proposed *PCBP* (0.79 s) is little larger than that of the *GLCM* (0.10s), it achieves much more efficient and robust classification performances for BUS images. Besides, *Ranklet* could sometimes achieve comparable classification results with the *PCBP*, however, it requires a much larger time cost (69.83 s). Even though the extraction of the *PCBP* includes a step of LBP-based calculation, it takes much less time than that of the *LBP* (212.80s). That is because the proposed *PCBP* is calculated in the simplest way (i.e., *P* = 8 and *R* = 1), as aforementioned in subsection ‘Local binary pattern (LBP)-based texture feature extraction’; whereas the *LBP* is calculated with *P* = 24 and *R* = 3 to achieve better classification performances, as described in Ref. [19]. Larger *P* and *R* make the *LBP* extraction procedure much more time-consuming than that of the *PCBP*. As a result, the average time cost of the *PCBP* is actually acceptable, considering its discrimination power for performing efficient and robust classification of BUS images.

## Discussions and conclusions

In this study, we proposed a novel phase-based texture descriptor, namely the phase congruency-based binary pattern for discriminating benign breast tumors from malignant ones in BUS images. The proposed PCBP is an oriented local texture descriptor, constructed by applying the local binary pattern based-method on oriented phase congruency images, which combines the advantages of both the phase congruency approach and the LBP method together.

The advantages of the PC approach mainly lie in two aspects. On one hand, the boundaries of breast tumors includes rich structural information to distinguish benign and malignant tumors, which is often extracted in morphological features after image segmentation, but rarely incorporated in texture features. Besides, internal echo patterns are also significant characteristics to differentiate benign and malignant tumors. Due to the use of local phase information to extract discontinuities (e.g., edges and corners) in BUS images, the abovementioned structural information can be well reflected on PC images, as shown in Figure 4. On the other hand, the phase congruency is invariant to variations of the image illuminations and contrast, which acts as a solid foundation for extracting robust texture features against gray-scale variations. As for the LBP, the most important properties are its tolerance regarding illumination changes and its computational simplicity [30], therefore it could efficiently describe various textural patterns in PC images to form the PCBP texture descriptor for the classification. Herein, the utilizing of both the PC approach and the LBP can well reinforce the classification efficiency and robustness of the proposed texture descriptor against illumination changes of BUS images caused by parameter adjustments in ultrasonic devices.

It is revealed in the experiments that the proposed PCBP texture descriptor achieves the best classification performance, evaluated by using both the LOO-CV method and the bootstrap method. Besides, a cross-contrast training/testing scheme is employed to verify the robustness of the texture descriptor against gray-scale variations of BUS images, and it is demonstrated in the experimental results that the proposed PCBP texture descriptor gets the highest AUC values and smallest variations, which suggests that the PCBP texture descriptor outperforms the Ranklet, the GLCM and the LBP. Additionally, the results of the statistical analysis further confirm the excellent performances of the PCBP for BUS image classification. Therefore, it can be concluded that the proposed PCBP texture descriptor is potentially useful in discriminate benign tumors from malignant ones in BUS images, and further helpful for breast ultrasound CADs.

The limitations of our work are twofold. Firstly, the ROIs used in our study are manually generated, which makes the CAD system not fully automatic and may introduce potential variations in ROI delineation process. Secondly, the size of the BUS image database is limited, which may have impact on experimental verifications. Since limitations exist, future work will be carried out for improvements. One is to apply lesion segmentation techniques to obtain ROIs automatically, thus minimizing the potential variations of manual delineation and making the CAD system more user-independent. The other is to establish a larger BUS image database, which will widen the case range and benefit our study. Finally, other effective textures based on multi-resolution and multi-orientation approaches, such as contourlet-based method [21], will also be considered in our future research.

## Abbreviations

BUS: Breast ultrasound
CAD: Computer-aided diagnosis
ROI: Region of interest
GLCM: Gray-level co-occurrence matrix
LBP: Local binary pattern
LBPV: LBP variance
PC: Phase congruency
PCBP: Phase congruency-based binary pattern
SVM: Support vector machine
ROC: Receiver operating characteristic
AUC: Area under ROC curve
LOO-CV: Leave-one-out cross-validation
